# Computer-Assisted Intramedullary Nailing of Intertrochanteric Fractures Did Not Prevent Tip–Apex Distance Outliers

**DOI:** 10.3390/jcm12237448

**Published:** 2023-11-30

**Authors:** Rasmus Holm Hansen, Jan Duedal Rölfing, Christian Lind Nielsen, Ole Brink, Per Hviid Gundtoft

**Affiliations:** 1 Department of Orthopaedics, Aarhus University Hospital, Palle Juul-Jensens Boulevard 99, DK-8200 Aarhus, Denmark; chlini@clin.au.dk (C.L.N.); olebrink@rm.dk (O.B.); pergun@rm.dk (P.H.G.); 2 Corporate HR, MidtSim, Central Denmark Region, Hedeager 5, DK-8200 Aarhus, Denmark

**Keywords:** hip fractures/diagnostic imaging, intramedullary fracture fixation, computer-assisted surgery, tip–apex distance, lag screw

## Abstract

Intertrochanteric femoral fractures are commonly treated with intramedullary nails (IMNs). A tip–apex distance (TAD) of more than 20–25 mm is associated with an increased risk of cut-out. The Stryker Adaptive Positioning System (ADAPT) is a computer-assisted navigation system designed to reduce TADs. We aim to assess if the ADAPT reduces the number of outliers with a TAD > 20 mm. All patients with intertrochanteric fractures treated with an IMN between 1 September 2020 and 12 March 2022 were included. Patients were included in three periods: a pre-ADAPT period (55 patients); an ADAPT period (50 patients), where it was compulsory to use the system; and a post-ADAPT period after the discontinuation of the system (59 patients). The TADs and lag screw protrusions beyond the lateral cortex were measured. The median TADs in the three periods were 17.0 mm (8–31 mm), 15.5 mm (9–30 mm), and 18.0 mm (11–32 mm), respectively. The absolute number of outliers with a TAD > 20 mm decreased from 15/55 patients in the pre-ADAPT period to 11/50 patients during the ADAPT period. This observation was not statistically significant, but this is likely due to the lack of power of the present study to show changes of this magnitude. However, our expectation that the ADAPT would diminish outliers markedly or close to zero outliers was not met, as we observed 11/50 = 22% outliers with a TAD > 20 mm when using computer-assisted surgery, i.e., ADAPT and Gamma3 for intertrochanteric fractures. Based on these findings, the use of the ADAPT was discontinued at our level 1 trauma center.

## 1. Introduction

Intertrochanteric fractures are among the most common fractures requiring surgery worldwide. The incidence in the US elderly population is approximately 350/100,000 inhabitants yearly [1]. Intertrochanteric fractures are often treated with intramedullary nailing [2]. One of the most common complications of intramedullary nailing is lag screw cut-out, which leads to rehospitalization and reoperations. Baumgaertner et al. found that an increased TAD was associated with lag screw cut-out and that the most optimal placement of the lag screw was center–center [3]. A TAD < 25 mm should be acceptable, although a study by De Brujin et al. suggests that a TAD < 20 mm is ideal [4]. To assist surgeons in placing the lag screw correctly, Stryker has developed the Adaptive Positioning Technology (ADAPT) (Stryker, Portage, MI, USA). The ADAPT system is a portable tracking and positioning system designed to customize and optimize implant positioning relative to the patient’s anatomy [5].

Previous studies on the ADAPT have shown promising results in terms of reducing the TAD, operating time, fluoroscopy time [6,7,8,9,10], and outliers [9]. A recent review article published in 2023 by Li et al. looked at clinical studies where the ADAPT was compared to the freehand method with regards to the placement of the lag screw [11]. Seven studies were included, with 326 fractures in the ADAPT group and 325 fractures in the freehand group. The ADAPT group showed a statistically smaller TAD and a better position of the lag screw than the freehand group. However, many of the studies differed from the conventional clinical setting in that they were performed with a single-surgeon setup [9], a trained computer operator, or another form of direct supervision [8,10]. Moreover, most studies that found that the ADAPT resulted in a reduction in the TAD did not show whether it reduced the number of outliers with a TAD above 20 or 25 mm. Therefore, studies on the usefulness of the ADAPT in a clinical setting are still needed.

The primary aim of this study was to assess whether the use of ADAPT entirely prevents or markedly reduces the number of outliers (TAD > 20 mm), when used in a clinical setting. A secondary aim was to assess whether the use of ADAPT reduces the lateral protrusion of the lag screw, the median TAD, the use of fluoroscopy, and duration of surgery.

## 2. Materials and Methods

In this prospective trial of the ADAPT system, all patients admitted to Aarhus University Hospital with AO type 31A2-31A3 intertrochanteric fractures were included in the period 1 September 2020–12 March 2022. To account for a potential learning period, we chose to have three periods: a pre-ADAPT period, an ADAPT period, and a post-ADAPT period. We planned to include approximately 50 patients in each period. Prior to the study, we agreed that if the ADAPT system could not reduce the number of outliers with a TAD > 20 mm after being used 50 times, we would discontinue its use in our department.

In the first period, the pre-ADAPT period (1 September 2020 to 27 February 2021), patients were operated on with the Stryker Gamma3 Hip Fracture Nailing System using the technique as described by the manufacturer without the use of computer assistance. In the following ADAPT period, 1 March 2021 to 30 September 2021, the ADAPT 2.1 system was introduced in our department, and it was mandatory for all surgeons to use the ADAPT system when nailing intertrochanteric hip fractures. The post-ADAPT period, 1 October 2021 to 12 March 2022, resembled the pre-ADAPT period, i.e., no ADAPT was available. In the pre- and post-ADAPT periods, we used regular equipment for intramedullary nailing with the Stryker Gamma3 intramedullary nail.

The following data were recorded for all patients in a database using Epidata v4.6.0.6 [12]: age, sex, AO type of fracture, nail length (short/long), fluoroscopy time (seconds), duration of surgery (minutes), surgeon level of education (intern, resident, fellow, attending), surgeon experience with ADAPT (yes/no), supervisor (yes/no), supervisor level of education (intern, resident, fellow, attending), supervisor experience with ADAPT (yes/no), presence of ADAPT expert (yes/no).

There were no exclusion criteria as all patients treated with a Gamma3 nail were considered eligible for operation using the ADAPT system.

All patients were scheduled for a three-month follow-up visit in the outpatient clinic, including a clinical examination and radiographic control.

**Figure 1:**
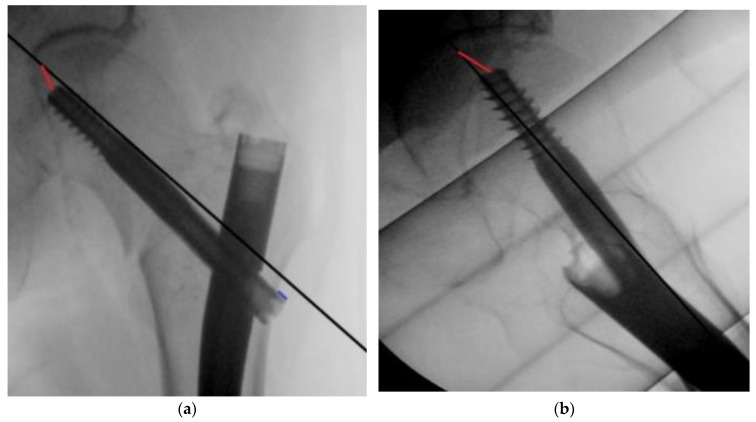
(**a**) Measuring the distance from lag screw tip to apex in the antero-posterior plane (red line) and the lag screw lateral protrusion (blue line). (**b**) Measuring the distance from lag screw tip to apex in the lateral plane (red line).

The primary outcome was the number of outliers, defined as a TAD > 20 mm and a TAD > 25 mm. The secondary outcomes were the lateral screw protrusion (Figure 1), median TAD, duration of surgery, and fluoroscopy time.

The TAD was measured based on perioperative fluoroscopy images using the method described by Baumgaertner et al. [3], where the distance from the screw tip to the apex of the femoral head in both the anteroposterior and lateral planes is used to generate a single numerical value for the TAD, calibrated by measuring the known diameter of the lag screw. The lateral screw protrusion was calculated in a similar manner (Figure 1).

More than 6 months after the last patient was included, we reviewed the patients’ medical files to determine the re-operation rate. We identified the cause of failure, indication for re-operation, and type of re-operation. The number of outliers with a TAD > 20 mm and >25 mm was compared between groups with a Chi square test. The median TAD and lag screw protrusion were analyzed using the Kruskal–Wallis test for non-parametric data with Dunn’s multiple comparison test. Data are presented as the median (min, interquartile range, max) or absolute numbers (%). Prism 9.4.1 for macOS was used for the statistical analyses and graphs.

The Regional Ethical Committee was consulted prior to commencing this study but stated that as the ADAPT system was introduced in our department as part of our standard care, no ethical approval was required and that the study was regarded as a quality improvement project.

## 3. Results

During the three periods, we included a total of 163 patients: for the pre-ADAPT, n = 55; for the ADAPT period, n = 50; and for the post-ADAPT, n = 59. Most of the patients were female (70%), and the mean age was 82 (range: 39 to 101) (Table 1). During the ADAPT period, 11 patients were excluded due to problems with the ADAPT version 2.1 hardware/software, i.e., the operation had to be performed without the ADAPT. In the pre- and post-ADAPT periods, all 55 and 59 consecutive patients were included in the final analysis.

**Figure 2:**
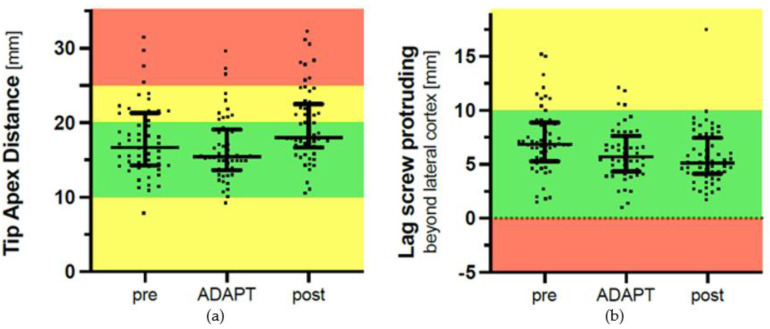
(**a**) Tip–apex distance (TAD). Pre-, during, and post-ADAPT periods in millimetres (upper panel). (**b**) Lag screw protrusion beyond the lateral cortex in millimetres (lower panel). Scatter plots with median and interquartile range given as error bars.

Regarding outliers, there were 15 out of 55 patients in the pre-ADAPT period with a TAD > 20 mm. For the ADAPT period, there were 11 out of 50 patients, and for the post-ADAPT period, there were 23 out of 59 patients. When comparing the ADAPT period to the pre- and post-ADAPT periods, the use of the ADAPT system did not reduce the number of outliers with a TAD > 20 mm (*p* = 0.10) and a TAD > 25 mm (*p* = 0.78). The main results of the study are depicted as scatter plots in Figure 2. The median TAD in the three periods was as follows: pre-ADAPT, 17.0 mm (8–31); ADAPT, 15.5 mm (9–30); and post-ADAPT, 18.0 mm (11–32). We found no reduction in the median TAD when using the ADAPT compared with the pre-ADAPT period (*p* = 0.62). However, the median TAD was higher in the post-ADAPT period compared to the ADAPT period (*p* = 0.001) (Table 1, Figure 2).

The ADAPT system did not significantly reduce the median protrusion of the lag screw of 5.7 (1–12) mm beyond the lateral cortex compared to the pre-ADAPT period (6.8 (2–15) mm, *p* = 0.15) and post-ADAPT period (5.1 (2–18) mm, *p* = 0.82).

There was no difference in the median fluoroscopy time when comparing the three periods (pre-ADAPT: 184 (62–786) seconds, ADAPT: 243 (112–532) seconds, post-ADAPT: 248 (86–506) seconds, *p* = 0.21).

We did not find a reduction in the surgery time in the ADAPT period compared to either the pre-ADAPT (*p* = 0.99) or post-ADAPT periods (*p* = 0.98).

Only one cut-out was observed in the follow-up period. This event occurred in a 94-year-old woman with an AO/OTA type A2.2 fracture that was treated with a long Gamma3 nail and combined usage of the ADAPT system with a TAD of 27 mm. The patient was reoperated with a total hip arthroplasty 52 days after the initial surgery.

## 4. Discussion

In this prospective trial of the ADAPT system, we included patients with intertrochanteric fractures in three periods: a pre-ADAPT period, where patients were operated on with the standard Gamma3 intramedullary nail technique (n = 55); an ADAPT period, where patients were operated on with the use of the ADAPT system (n = 50); and a post-ADAPT period, where patients were operated on without the use of the ADAPT system (n = 59). The ADAPT system did not prevent outliers entirely. In fact, the number of outliers was still 11/50 = 22% when using the system. This reduction from 15/55 = 27% in the pre-ADAPT period was not statistically significant. The lateral protrusion of the lag screw was not significantly different between the three periods (*p* = 0.15–0.82). Similarly, there were no significant differences in the duration of surgery or fluoroscopy time (*p* = 0.21).

We did not find a significant reduction in outliers when comparing the ADAPT period to the pre- and post-ADAPT periods for outliers with a TAD > 20 mm (*p* = 0.10) or for outliers with a TAD > 25 mm (*p* = 0.78). This is in line with the randomized controlled trial performed by Lilly et al. [13], who likewise found no difference in the TAD, but it is in contrast to the study by Kuhl et al., who found that the ADAPT improved the median TAD [9]. This dissimilarity in results could be due to the difference in study design, as Kuhl et al. compared a period without the ADAPT to a period with the use of the ADAPT over an 8-year study period, with all surgeries performed by a single surgeon. Therefore, the decreased median TAD and reduced number of outliers in Kuhl et al.’s study could be due to the learning curve with the Gamma3 nail in general. The study by Lilly et al. only included patients operated on by fellowship-trained surgeons, who may have the skill level to achieve a satisfactory TAD regardless of technique and helping devices. This is probably also why we did not find a reduction in the number of outliers. Although most of the surgeons in this study were younger doctors, they were supervised by experienced surgeons, who most likely have the capability to acquire an acceptable TAD. Furthermore, the Gamma3 nail technique already has additional adjuncts to improve the placement and TAD of the Gamma3 nail, including the one-shot device. Therefore, the lack of a reduction in outliers when using the ADAPT might be because the Gamma3 nail is already developed to a stage where most surgeons will obtain satisfactory results without any additional adjuncts. Whether the outliers in the ADAPT period can be attributed to incorrect use of the ADAPT, a technical error of the ADAPT, or a disregard of the feedback the surgeon provided for the ADAPT intraoperatively is not possible to determine in this study.

The ADAPT visualizes the placement and length of the lag screw. Therefore, it could potentially assist the surgeon in determining the correct length of the lag screw, with a decreased risk of lateral protrusion and a lower risk of lateral hip pain. This has not previously been studied, and we did not find a significant decrease in lateral protrusions when using the ADAPT. The reason for this finding is most likely similar to the reasons why the ADAPT failed to reduce the number of outliers and the median TAD.

We found a statistically significantly lower median TAD when comparing the ADAPT period (median TAD = 15.5 mm) with the post-ADAPT period (median TAD = 18.0) (*p* = 0.001) but not when comparing the ADAPT period with the pre-ADAPT period (median TAD = 17) (*p* = 0.62). As the median TAD in all three periods was below 20 mm, this reduction in the TAD might be statistically significant, but it is not clinically relevant.

In a study by Hestehave et al., some surgeons suggested that even though they did not find the ADAPT useful, it could serve as a teaching tool for less experienced surgeons [14]. However, in this study, the TAD did not decrease following the ADAPT period; rather, it increased, although not significantly. Therefore, we conclude that ADAPT’s ability as a teaching tool might be diminished. Training for guidewire placement for the nail entry point and lag screw can be conducted safely using simulation-based training [15,16,17,18]. Improving intraoperative performance through these training modalities may be more effective in achieving optimal implant positioning than computer-assisted nailing.

The strength of this study is the setting in which the ADAPT was used by several different surgeons on all patients treated with the Gamma3 nail during a specific period, thereby mimicking the real world in which ADAPT might be used.

One limitation to our study is that the usage of the ADAPT was mandatory during the ADAPT period, which may have forced some experienced surgeons to use a system and operative technique that they were not familiar with. Moreover, these experienced surgeons might not benefit from the ADAPT, as they were probably more likely to achieve acceptable results for the TAD beforehand. We found it necessary to make it mandatory as the system had already been available for surgeons for six months without being used. Lastly, we did not stratify the analysis based on surgical experience because consultants operated on a consistently low number of cases, i.e., 6/55 (11%) pre-ADAPT, 6/50 (12%) ADAPT, and 8/59 (14%) post-ADAPT. Moreover, the ADAPT is designed to be an adjunct for all surgeons.

A second limitation to our study is the lack of a power calculation. We did not perform a power calculation prior to this study, as we beforehand agreed that if the ADAPT system did not reduce the number of outliers significantly when used on 50 patients, we would discontinue its use in our department.

Based on the data regarding outliers (TAD > 20 mm; TAD > 25 mm) provided in Table 1, the following post hoc power calculations can be performed in order to inform colleagues to perform adequately sized studies in the future.

With a TAD > 20 mm, the combined pre-ADAPT and post-ADAPT rate of outliers was (11 + 4 + 15 + 8)/(55 + 59) = 38 outliers/114 patients = 33%, while the corresponding rate during the ADAPT-period was (8 + 3)/50 = 22%. Based on these findings, the post hoc power of the study (alpha = 0.05) was only 28%. Consequently, a sample size calculation with a power of 0.8, an alpha of 0.05, and group incidences of 33% and 22% would require 257 patients in each group. In this light, the study can be argued to be underpowered.

However, the advantage of the ADAPT was thought to be to reduce the outliers completely, i.e., a reduction from approximately a 25–27% rate of outliers (TAD > 20 mm) to 0%. In this case, only 42 patients would be needed in each group to have a power of 0.95 with an alpha of 0.05. The current study revealed that the ADAPT did not eliminate outliers completely. On the contrary, our study documents that outliers were still common despite the use of the ADAPT, i.e., 11/50 = 22%. Therefore, our initial hope and belief that computer-assisted surgery could entirely prevent or markedly reduce the number of outliers was not confirmed. Moreover, neither residents nor consultants liked to use the system [14]. We therefore decided to discontinue the use of the ADAPT system at our institution.

### Perspectives

Computer-assisted fracture surgery is on the rise and has been extensively investigated in recent years [19,20]. Adequate reduction and optimal implant positioning play a crucial role in achieving stability and promoting successful healing outcomes. Computer-assisted techniques may be used during planning but also during fracture surgeries when reducing the fracture or accurately placing implants [21,22,23]. However, the clinical benefit of computer-assisted fracture surgery is a matter of debate, and several authors highlight that the level of evidence in studies advocating computer-assisted surgery is low [24,25]. The role of computer-assisted surgery in other related orthopedic procedures like total knee replacement and high tibial osteotomies has been thoroughly investigated. While some studies highlight advantages such as improved accuracy, others report the failure of computer-assisted surgery to reduce the number of outliers [26,27]. In computer-assisted total knee arthroplasty, an outlier rate of 10% has been reported [28]. This rate is lower than the 22% outlier rate of the ADAPT system in our present study but can also serve as an example that the idea of computer-assisted surgery preventing outliers altogether may be unrealistic in the near future. Theoretically and ideally, computer-assisted surgery should narrow the bell curve, i.e., concentrate the results around the mean and thus prevent outliers. Nonetheless, recent and ongoing developments in computer-assisted fracture surgery and augmented reality may overcome this current limitation in the future.

## 5. Conclusions

In the present study, TAD outliers (>20 mm) were still present in 11/50 = 22% of hip fracture patients being treated with the use of computer-assisted surgery (ADAPT for Gamma3 hip fracture nailing system). Based on these findings, the use of ADAPT was discontinued at our level 1 trauma center.

## Figures and Tables

**Table 1:** Demographics and results.

	Pre-ADAPT n = 55	ADAPT n = 50	Post-ADAPT n = 59
Female/male Age (years) BMI (kg/cm^2^) ASA	40/15 83 (39, 73–88, 101) 23 (18, 21–27, 37) 2 (1, 2–3, 4)	36/14 82 (45, 70–89, 97) 23 (15, 21–26, 34) 3 (1, 2–3, 4)	39/20 82 (52, 74–87, 99) 24 (15, 22–27, 37) 3 (1, 2–3, 4)
Nail (short/long) Procedure time (minutes) Median fluoroscopy time (second)	31/24 64 (16, 46–100, 242) 184	27/23 74 (23, 58–90, 211) 243	27/32 69 (19, 45–97, 167) 248
TAD (mm) TAD < 20 mm TAD 20–25 mm TAD > 25 mm	16.7 (8, 14–21, 32) 40 (73%) 11 (20%) 4 (7%)	15.4 (9, 14–19, 30) 39 (78%) 8 (16%) 3 (6%)	18.0 (11, 17–23, 32) * 36 (61%) 15 (25%) 8 (14%)
Lag screw lateral protrusion (mm)	6.8 (2, 5–9, 15)	5.7 (1, 4–8, 12)	5.1 (2, 4–7, 17)
Surgeon experience (resident/consultant)	49/6	44/6	51/8

Data are either presented as median (minimum, interquartile range, maximum) or absolute number (%). * *p* < 0.05 ADAPT vs. post-ADAPT, i.e., there is only one statistically significant difference in the table.

## Data Availability

The data presented in this study are available on request from the corresponding author. The data are not publicly available due to restrictions from our department and in accordance with Danish data registration.
